# Complicated bilateral fungal emphysematous pyelonephritis in a 55-year-old diabetic female: A case report and review of literature

**DOI:** 10.18502/cmm.4.2.61

**Published:** 2018-06

**Authors:** Ensieh Lotfali, Sara Abolghasemi, Fatemeh Sadat Gatmirimotahhari, Mohammad Alizadeh, Zahra Arab-Mazar

**Affiliations:** 1 Department of Medical Parasitology and Mycology, School of Medicine, Shahid Beheshti University of Medical Sciences, Tehran, Iran; 2 Infectious Diseases and Tropical Medicine Research Center, Shahid Beheshti University of Medial Sciences, Tehran, Iran

**Keywords:** Candida albicans, Emphysematous pyelonephritis

## Abstract

**Background and Purpose:**

Emphysematous pyelonephritis (EPN) is a rare and serious disease causing acute renal failure. Diabetes is a major risk factor for this infection.

**Case report:**

Herein, we present the case of a 55-year-old female patient with diabetes and EPN caused by *Candida albicans*. The infection was complicated with endophthalmitis and endocarditis. The results of antifungal susceptibility analysis showed that *C. albicans* was resistant to fluconazole and susceptible to amphotericin-B and itraconazole. Infection could be controlled by amphotericin-B followed by itraconazole therapy, and the patient was discharged in good condition while receiving antifungal therapy.

**Conclusion:**

Complicated pyelonephritis with unusual microorganisms should be considered in patients with diabetes and urinary symptoms.

## Introduction

Candiduria can be a sign of colonization, urinary tract infection, or severe systemic candidiasis. Immunocompromised hosts and patients with indwelling catheters are often at risk for symptomatic candiduria [1]. 

Emphysematous pyelonephritis (EPN) is a rare necrotizing infection of the kidneys that predominantly affects middle-aged women with diabetes [2]. EPN is usually caused by glucose-fermenting bacteria; the most common etiologic agents are *Escherichia coli*, *Klebsiella pneumonia*, and *Proteus mirabilis*. In addition, some cases of infection with *Clostridium* spp., *Candida* spp., *Aspergillus* spp., and EPN have been reported [3]. Patients with EPN initially show relatively vague symptoms, including fever, dysuria, hematuria, and abdominal pain, but frequently undergo a sudden deterioration in symptoms such as depressed consciousness and shock [4]. 

**Figure 1:**
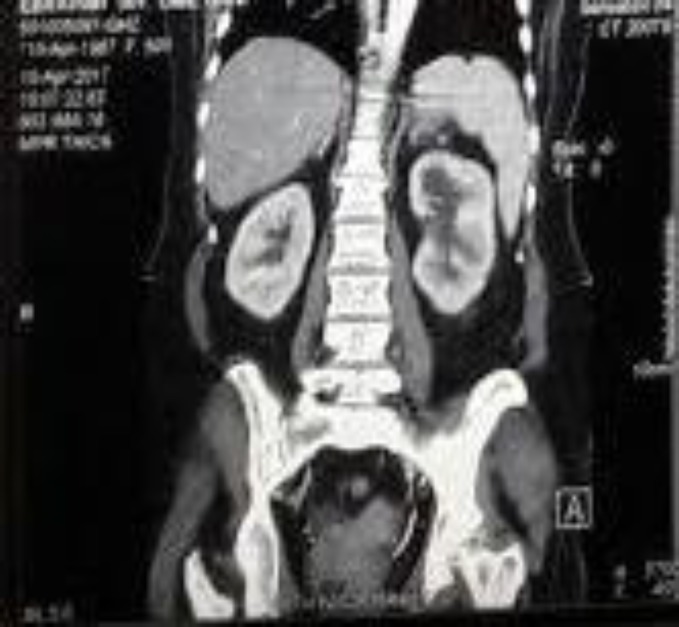
Abdominal and pelvic computed tomography, gas bubble in both renal parenchyma and surrounding fat stranding

A 55-year-old female diabetic patient was referred to Labbafi Nejad Tertiary Care Center in Tehran, Iran, with complaints of fever, anorexia, weakness, nausea, vomiting, and flank pain; she had been admitted to another hospital 10 days earlier. Her illness had started 15 days earlier with darkening of urine color and frequency and urge incontinency followed by fever, general weakness, nausea, vomiting, and flank pain. Glaucoma and some episodes of urinary tract infection were noted in her medical history. Abdominal and pelvic computed tomography (CT) scan elicited the presence of gas bubbles in both renal parenchyma and surrounding fat stranding compatible with bilateral EPN (Figure 1) also gas bubbles in the lumen of bladder compatible with emphysematous cystitis; an echoic lesion suggestive of renal stone in the right lower calice was also reported. 

Due to moderate hydronephrosis in the left kidney, ureteral double-J (DJ) catheter was inserted. She had received intravenous (IV) ceftriaxone 2 g/day and due to lack of response to therapy was referred to our hospital. On admission, she was febrile with oral temperature of 39°C with stability in other vital signs. Physical examination was unremarkable except the paleness, mild dehydration, and bilateral costovertebral angle (CVA) tenderness. Peripheral blood leukocyte count was 18,300/µL with 75% neutrophil; hemoglobin and platelet counts were 9.6 mg/dL and 465 ×10^9^/L, respectively. Renal function tests revealed 62 mg/dL urea and 1.64 mg/dL creatinine. The first urine analysis showed decrease in specific gravity (SG = 1030), hematuria (1+), proteinuria (2+), glycosuria (3+) and pyuria, moderate bacteriuria, and many yeasts. Urine and blood samples were sent for microbiological examinations. 

The patient started empiric therapy with IV imipenem. Urologic consultation for surgical intervention considered the continuation of IV antibiotic therapy without surgery; after 48 hours, the patient was still febrile with hypotension and her blood gas showed metabolic acidosis. Vancomycin, colistin, and fluconazole were added to empiric antibiotic regimen [5] and DJ catheter was removed.

The yeasts isolated from the urine culture (U/C) were subcultured on Sabouraud agar (Merck, Germany) and incubated at 37°C for 2-3 days. U/C yielded *Candida* spp. Blood samples were inoculated into the biphasic standard blood culture bottles (BBHI) containing brain-heart infusion agar (BHIA)/brain-heart infusion broth (BHIB) (PadtanTeb Co. Tehran, Iran) and incubated at 37°C. After 72 hours, the positive blood cultures were read. Blood culture results revealed contamination with *Candida* spp. and *Enterococcus facium*. The isolated *Candida* spp. were cultured on CHROMagar Candida medium (bioMérieux, France) and incubated at 37°C for 48 hours; then, *C. albicans* was identified based on green color grown colonies. Antifungal susceptibility testing was performed for the isolated *C. albicans* against amphotericin-B, fluconazole, and itraconazole, all purchased from Sigma-Aldrich, USA, according to the Clinical and Laboratory Standards Institute (CLSI, document M27-S3) guidelines [6].

Minimum inhibitory concentration (MIC) against amphotericin-B was ≤1 µg/mL, which is considered as susceptible and >1 µg/mL as resistant. Also, regarding itraconazole, MICs ≤ 0.125 and ≥1 µg/mL and for fluconazole, MICs ≤ 2 and ≥ 8 µg/mL were considered as susceptible and resistant, respectively. The antifungal susceptibility analysis showed that *C. albicans* isolates were resistant to fluconazole and susceptible to amphotericin-B and itraconazole [7]. Antibiotic susceptibility testing for enterococci was performed by E-test and the results were as follows: resistant to ampicillin, vancomycin, imipenem, gentamicin, and streptomycin [8]. Due to candidemia, ophthalmologic examination was performed, which indicated inflammation of the anterior and posterior segments of both eyes and Candida retinitis of the right eye; two intravitreal (IV) injections of amphotericin-B 5 μg in 0.1 mL distilled water were administered at a 48-hour interval, and IV fluconazole was replaced with liposomal amphotericin-B 3 mg/kg. Vancomycin was switched to IV linezolid 600 mg twice daily and the requested transesophageal echocardiogram (TEE) demonstrated endocarditis with two vegetations on the anterior mitral valve leaflet (4 × 1.8 mm) and the anterior leaflet of tricuspid valve (6 × 7mm). 

**Table 1:** Overview of 5 reported articles of fungal emphysematous pyelonephritis (1986-2012).

	**Year/age/ sex**	**Location**	**Agent**	**Underlying disease**	**Clinical presentations**	**Examination**	**Treatment**	**Ref.**
**1**	1986/51/M	USA	*C. albicans*	diabetes mellitus	Nausea, vomiting and flank pain	Urine culture	NI	[17]
**2**	1999/74/M	Germany	*C. tropicalis and C. glabrata*	diabetes mellitus	Stuporous mental condition	Urine culture and serologic tests	AMB	[18]
**3**	2004/-/-	NI	*C. tropicalis*	NI	Recurrent hematuria, flank pain, acute fulminant renal failure, and obstruction by a sloughed papilla	NI	NI	[19]
**4**	2005/43/F	Malaysia	*C. albicans*	Diabetes mellitus	Severe pain in the left lumbar region associated with high-grade fever, chills and rigors	Urine culture	FLU and nephrectomy	[20]
**5**	2012/60/F	NI	*C. parapsilosis*	Diabetes mellitus	Febrile illness associated with abdominal pain	Urine culture	AMB	[21]

NI =Not indicated; *C., Candida*; FLU, fluconazole; AMB, Amphotericin B; Ref., Reference

Her cardiothoracic surgery consultation suggested high mortality risk for cardiac surgery. Therefore, suppressive therapy was continued for Candida endocarditis. Repeated blood cultures remained negative; she completed the recommended six-week course of antibiotic therapy consisting of IV linezolid and liposomal amphotericin-B from the first negative blood culture; patient symptoms improved gradually. Inflammatory markers such as estimated sedimentation rate and C-reactive protein decreased. Hence, amphotericin-B was replaced with itraconazole 200 mg twice a day and the patient was discharged in good condition while receiving long-term antifungal therapy.

On six-week follow-up, repeated TEE demonstrated decreased vegetation size and then, complete resolution, and follow-up ophthalmologic exams six weeks and three months after therapy showed improvement of endophthalmitis. 

## Discussion

Emphysematous pyelonephritis is a necrotizing and mostly unilateral renal infection. There are few case reports of bilateral EPN. The radiological classification of EPN based on the extent of gas is described in literature as follows: stage I, gas within the renal parenchyma or the perinephric tissues; stage II, the presence of gas in the kidney and its surroundings; and stage III, extension of gas through Gerota's fascia or presence of bilateral EPN [9]. The remarkable difference of the current case from other reports was the type of infectious agent. Fungi are a rare cause of emphysematous pyelonephritis. Several case reports are published on patients with fungal EPN, but with the unilateral form [8].

Candida pyelonephritis may cause candidemia and sepsis. According to different reports, fever and candiduria are the most predominant primary symptoms pertaining to Candida pyelonephritis [7]. The mortality of invasive candidiasis may be 40-60% [10].

Our patient had diabetes with candiduria and urinary symptoms; she did not have any other sources for candidemia such as central vein catheter, severe mucositis, total parenteral nutrition (TPN) therapy, and abdominal surgery with leakage of anastomosis; hence, it is reasonable to conclude that the patient had candidemia from a urinary tract source. To ensure the accuracy of the positive results, urine culture should be repeated. The presence of candiduria in adults was considered ≥ 10^4^ CFU/ml urine [11]. The available azoles such as voriconazole and posaconazole have minimal excretion of the active compound into urine and as a result, they are not used for the treatment of such infections [1]. 

According to the guidelines of the Infectious Diseases Society of America (IDSA), in symptomatic pyelonephritis, amphotericin-B deoxycholate is an alternative treatment [12]. In recent years, the nosocomial pathogenicity of *Enterococcus *spp. emerged and due to the development of resistance to many antimicrobial agents, it caused great concern. *Enterococcus *spp*.* are major nosocomial pathogens. In most of the cases, positivity of *Enterococcus *spp*.* in blood cultures represents true infection, and only 10-15% may be a contamination [13]. In different studies, the percentage of endocarditis, as the cause of enterococcal bacteremia, vary (1% to 32%) [14]. 

Candida endocarditis, which is the most serious form of infective endocarditis, occurs in candidemia cases and remains with a high mortality rate of about 50%. *C. albicans* is accountable for 24-46% of all the cases of fungal endocarditis, with a mortality rate of 46.6-50% [15].

Based on the latest IDSA guideline for native valve endocarditis, lipid formulation of amphotericin-B, 3–5 mg/kg daily, with/without flucytosine, can be the treatment of choice and should be continued for six weeks after valve replacement, but in patients for whom surgery is contraindicated, long-term suppression is recommended and due to the risk of relapse, follow-up for several years after treatment should be considered [12].

In the current case, both microorganisms detected in blood cultures (i.e., *Candida* and *Enterococous *species) could be the cause of endocarditis; therefore, the patient received both treatments. According to the guidelines, lumbar puncture and brain imaging are not recommended for adult patients with candidemia and no central nervous system (CNS) symptoms [16].

Chorioretinitis and endophthalmitis are of the major complications of candidemia. Ophthalmological examination should be performed for all candidemia patients. Liposomal amphotericin-B, 3–5 mg/kg IV daily, with or without oral flucytosine, is recommended to treat fluconazole- and voriconazole-resistant isolates, and for patients with macular involvement, intravitreal injection of either amphotericin-B deoxycholate, 5–10 μg/0.1 mL distilled water, or voriconazole, 100 μg/0.1 mL distilled water or normal saline should be administered. The duration of treatment is 4-6 weeks and depends on resolution of the lesions in serial ophthalmologic visits [12]. There were some case reports regarding bilateral fungal emphysematous pyelonephritis.

## Conclusion

Due to the increasing prevalence of infection with fluconazole-resistant *C. albicans* and the risk of complicated pyelonephritis in patients with diabetes, these infections should be considered in differential diagnostic measures in diabetic patients. 
